# Supplementary material for: Corticotropin-Releasing Hormone: A Novel Stimulator of Somatolactin in Teleost Pituitary Cells
Source: Cells. 2023 Dec 5;12(24):2770. doi: 10.3390/cells12242770 (PMC10741825; doi:10.3390/cells12242770)
Supplement: Supplementary file 1 [file cells-12-02770-s001.zip › cells-2697764 supplementary.pdf]

Supplemental Table S1. Background information for test substances used in this study

Test substance	Biological action	Vendor/Supplier	Cat. No.
MDL12330A	AC inhibitor	Merck	444200
H89	PKA inhibitor	Calbiochem	371963
GF109203X	PKC inhibitor	Sigma	B6292
U73122	PLC inactivator	Sigma	U6756
2-APB	IP3 receptor blocker	Calbiochem	100065
Nifedipine	VSCC blocker	Sigma	N-7634
Calmidazolium	CaM antagonist	RBI	C-100
KN62	CaMK-II blocker	Calbiochem	422706

Supplemental Table S2. Primers used for tissue expression profiling and real-time PCR.

Target	Forward primer	Reverse primer	Length	Annealing Tm
**Primers for tissue distribution**				
CRH1a	GGCTCGGTAACAGAAACCAGAA	GTCAGGTCCAGGGAAATCG	223 bp	59 ℃
CRH1b	GGATGGACAACACCGCTCACA	CGACACTACCGACCTTGCCTC	249 bp	58 ℃
UTS1	CTGACCACCCACATCCCCCT	AGTTCCGCCTGTTCCCTTTG	362 bp	63 ℃
CRHR1	TTACACTAATCTCCAGGGCAACA	ACATTATTGGTGGTGTTGTATCG	250 bp	62 ℃
CRHR2	CCTTCGCAACATAATCCACT	AGATAGCAGCCCTCCACAAACA	292 bp	60 ℃
**qPCR primers**				
SLα	ACCCACTGTACTTCAATCTCC	CGTCGTAACGATCAAGAGTAG	283 bp	52 ℃
SLβ	TGTTTGAGGAGATGCTCGTTT	CCACCGTCACCCAATATCTGT	310 bp	60 ℃
β-actin	CTGGTATCGTGATGGACTCT	AGCTCATAGCTCTTCTCCAG	280 bp	56 ℃

Supplemental Table S3. Antibody table for antibodies used in IHS and ELISA for carp pituitary hormones

Protein Target	Antigen & Source of Sequence Information	Name of Antibody	Name of individual providing the antibody	Species for raising antibody/application	RRID No.
Carp SLα	Recombinant carp SLα (GenBank number EF372074.1)	Antiserum against carp SLα	Prof AOL Wong, Univ of Hong Kong, Hong Kong	polyclonal in Rabbit / IHS, RIA, ELISA & WB	AB_2631193
Carp SLβ	Recombinant carp SLβ (GenBank number EF372075.1)	Antiserum against carp SLβ	Prof AOL Wong, Univ of Hong Kong, Hong Kong	polyclonal in Rabbit / IHS, RIA, ELISA & WB	AB_2631194

(Abbreviations:SLα, Somatolactin α; SLβ, Somatolactin β; IHS, Immuno-histochemical Staining; ELISA, Enzyme-linked NImmunosorbent Assay; WB, Western blot)

Supplemental Figure S1

(A)

```
                    Signal Peptide
Grass carp  CRH1a   MKLHVLLAAVTLGVHVSLS-ESAPGHTDTQLP----------------------------   31
Zebrafish   CRH1a   MNLHVVVVAVSLGLSVWSS-DCAPGHTDAPLP----------------------------   31
Carassius   CRH1a   --------------------ECRAIESSSNQP----AADPD------------GERQAP-   23
Frog        CRH     MKFQLWVSTGILLVSLLPCHECRAFI-KSPAS----SPGAL-----LPAL---SNSQ---   44
Chicken     CRH     MKLQPLVCAGILLLALLPCHECRALS-KSPGA----ARGALQQPDFFPQQ---QQQQQQQ   52
Mouse       CRH     MRLRLLVSAGMLLVALSSCLPCRALLSRGSVP---RAPRAPQPLNFLQP----EQPQQPQ   53
Human       CRH     MRLPLLVSAGVLLVALLPCPPCRALLSRGPVPGARQAPQHPQPLDFFQPPPQSEQPQQPQ   60

Grass carp  CRH1a   ---FLMRLGEEYFFRMDNTAHSPRAPERRSPSRT-----EALQLQLTQR--------LLG   75
Zebrafish   CRH1a   ---VLMRVGEEYRIRLDN---SPSALDRKAPSR--------IQLQLTQR--------LSG   69
Carassius   CRH1a   --PVLARIGEEYFIRLGNRDQNPLRSPADSFPETSQYSKRALQLQLTQR--------LLE   73
Frog        CRH     --PFLLRMGEEYFLRLGNLHKHSPGSFPEASAGNFVRAVQQLQAQQWSS--------QPG   94
Chicken     CRH     TLPVLLRMGEEYFLRLGHLTKRPAGPFSASSGG-------HLRPEASAE--------LLR   97
Mouse       CRH     --PVLIRMGEEYFLRLGNLNRSPAARLSPNSTPLTAGRGSRPSHDQAAANFFRVLLQQLQ  111
Human       CRH     ARPVLLRMGEEYFLRLGNLNKSPAAPLSPASSLLAGGSGSRPSPEQATANFFRVLLQQLL  120

                                                      CRH1a
Grass carp  CRH1a   GKVGSVGN-------------GIMNSFVERERRSEDPPISLDLTFHLLREVLQMARAEQL  122
Zebrafish   CRH1a   GQIGGVGN-------------GILSSPEDRERRSEDPPISLDLTFHLLREVLQMARAEKM  116
Carassius   CRH1a   GKVGNIGR------LDGNYALR-ALDSVERERRSEEAPISLDLTFHLLREVLEMARAEQM  126
Frog        CRH     MRAASLDG------ADSPYSAQ--EDPTEKAKRAEEPPISLDLTFHLLREVLEMARAEQI  146
Chicken     CRH     AAAAQLQG------SGSPEGDEGAGEAVEREKRSEEPPISLDLTFHLLREVLEMARAEQL  151
Mouse       CRH     MPQRSLDSRAEPAERGAEDALGGHQGALERERRSEEPPISLDLTFHLLREVLEMARAEQL  171
Human       CRH     LPRRSLDSPAALAERGARNALGGHQEAPERERRSEEPPISLDLTFHLLREVLEMARAEQL  180

Grass carp  CRH1a   AQRASNNRKIMDDLGK*   138
Zebrafish   CRH1a   TQRVTHNRKIMDDLGK*   132
Carassius   CRH1a   AQQAHSNRKMMEIFGK*   142
Frog        CRH     AQQAHSNRKLMDIIGK*   162
Chicken     CRH     AQQAHSNRKLMEIIGK*   167
Mouse       CRH     AQQAHSNRKLMEIIGK*   187
Human       CRH     AQQAHSNRKLMEIIGK*   196
```

(B)

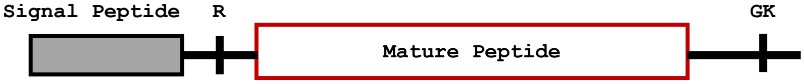


```
Grass carp  CRH1a   SEDPPISLDLTFHLLREVLQMARAEQLAQRASNNRKIMDDL-NH2
Zebrafish   CRH1a   SEDPPISLDLTFHLLREVLQMARAEKMTQRVTHNRKIMDDL-NH2
Carassius   CRH1a   SEEAPISLDLTFHLLREVLEMARAEQMAQQAHSNRKMMEIF-NH2
Frog        CRH     AEEPPISLDLTFHLLREVLEMARAEQIAQQAHSNRKLMDII-NH2
Chicken     CRH     SEEPPISLDLTFHLLREVLEMARAEQLAQQAHSNRKLMEII-NH2
Mouse       CRH     SEEPPISLDLTFHLLREVLEMARAEQLAQQAHSNRKLMEII-NH2
Human       CRH     SEEPPISLDLTFHLLREVLEMARAEQLAQQAHSNRKLMEII-NH2
```

Supplemental Figure S1. Sequence alignment of CRH1a. (A) Amino acid sequence alignment of CRH1a in different species. (B) Comparison of CRH mature peptide sequences in different species.

Supplemental Figure S2

(A)

```
                       |-- Signal Peptide --|
Grass carp CRH1b MKLNFLVTTVALLVAFPPPYECRAIESSSNQP----ATDPD------------GERQSP-    43
Zebrafish  CRH1b MKLNFLVTTVALLVAFPPPYECRAIESSFNQP----AADPD------------GERQSP-    43
Frog       CRH   MKFQLWVSTGILLVSLLPCHECRAFI-KSPAS----SPGAL-----LPAL---SNSQ---    44
Chicken    CRH   MKLQPLVCAGILLLALLPCHECRALS-KSPGA----ARGALQQPDFFPQQ---QQQQQQ    52
Mouse      CRH   MRLRLLVSAGMLLVALSSCLPCRALLSRGSVP---RAPRAPQPLNFLQP----EQPQQPQ   53
Human      CRH   MRLPLLVSAGVLLVALLPCPPCRALLSRGPVPGARQAPQHPQPLDFFQPPPQSEQPQQPQ   60

Grass carp CRH1b --PVLARLGEEYFIRLGNRNQNSLRSPADSFSETSQYSKRALQLQLTQR---------LLE  93
Zebrafish  CRH1b --PVLARLGEEYFIRLGNRNPTSPRSPADSFPETSQYPKRALQLQLTQR---------LLE  93
Frog       CRH   --PFLLRMGEEYFLRLGNLHKHSPGSFPEASAGNFVRAVQQLQAQQWSS---------QPG  94
Chicken    CRH   TLPVLLRMGEEYFLRLGHLTKRPAGPFSASSGG-------HLRPEASAE---------LLR  97
Mouse      CRH   --PVLIRMGEEYFLRLGNLNRSPAARLSPNSTPLTAGRGSRPSHDQAAANFFRVLLQQLQ  111
Human      CRH   ARPVLLRMGEEYFLRLGNLNKSPAAPLSPASSLLAGGSGSRPSPEQATANFFRVLLQQLL  120

                                                  ' |-------- CRH1b ---------
Grass carp CRH1b GKVGNIGR------LDGNYALR-ALDSMERERRSEEPPISLDLTFHLLREVLEMARAEQM  146
Zebrafish  CRH1b GKVGNIGR------LDGSYALR-ALDSMERERRSEEPPISLDLTFHLLREVLEMARAEQM  146
Frog       CRH   MRAASLDG------ADSPYSAQ--EDPTEKAKRAEEPPISLDLTFHLLREVLEMARAEQI  146
Chicken    CRH   AAAAQLQG------SGSPEGDEGAGEAVEREKRSEEPPISLDLTFHLLREVLEMARAEQL  151
Mouse      CRH   MPQRSLDSRAEPAERGAEDALGGHQGALERERRSEEPPISLDLTFHLLREVLEMARAEQL  171
Human      CRH   LPRRSLDSPAALAERGARNALGGHQEAPERERRSEEPPISLDLTFHLLREVLEMARAEQL  180

                     ------------|''
Grass carp CRH1b AQQAHSNRKMMEIFGK*   162
Zebrafish  CRH1b AQQAHSNRKMMEIFGK*   162
Frog       CRH   AQQAHSNRKLMDIIGK*   162
Chicken    CRH   AQQAHSNRKLMEIIGK*   167
Mouse      CRH   AQQAHSNRKLMEIIGK*   187
Human      CRH   AQQAHSNRKLMEIIGK*   196
```

(B)

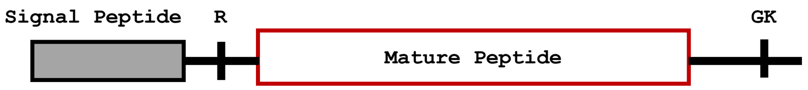


```
Grass carp CRH1b SEEPPISLDLTFHLLREVLEMARAEQMAQQAHSNRKMMEIFGK-NH2
Zebrafish  CRH1b SEEPPISLDLTFHLLREVLEMARAEQMAQQAHSNRKMMEIFGK-NH2
Frog       CRH   AEEPPISLDLTFHLLREVLEMARAEQIAQQAHSNRKLMDIIGK-NH2
Chicken    CRH   SEEPPISLDLTFHLLREVLEMARAEQAQQAHSNRKLMEIIGK -NH2
Mouse      CRH   SEEPPISLDLTFHLLREVLEMARAEQLAQQAHSNRKLMEIIGK-NH2
Human      CRH   SEEPPISLDLTFHLLREVLEMARAEQLAQQAHSNRKLMEIIGK-NH2
```

Supplemental Figure S2. Sequence alignment of CRH1b. (A) Amino acid sequence alignment of CRH1b in different species. (B) Comparison of CRH1b mature peptide sequences in different species.

Supplemental Figure 3

(A)

```
                            ├ Signal Peptide ┤
Grass carp UTS1   MKPVPLVLLITSVLLTTPIPPDTCRPRDLSLMSNQVDDVLLTRAG---DDAMLYLVGEKL   57
Zebrafish  UTS1   MKPVPLVLLITSVLLS-HIPLSFCRPRDLNSLHNQLDDLLLGRPGGGGGDAMSVLMGEKL   59
Goldfish   UTS1   MKPVPLVLLITSVLLTTHIPLSTCRPRDLSLVNSQLDDVLSNGAG---DDAMSYLVGEKL   57
Frog       UCN1   MKTALLTLLTCYLILTAYTGARPFARSMFDSHQLGLNGLLGDENT----QALLYFLREKI   56
Chicken    UCN    MRR----ALLTVLLLLCRPFPAAPR--PARPD-----------------------------   26
Mouse      UCN    MIQRGRATLLVALLLLAQLRPESSQWSPAAAAATGVQDPNLRWSP---------------   45
Human      UCN1   MRQAGRAALLAALLLLVQLCPGSSQRSPEAA---GVQDPSLRWSP---------------   42

Grass carp UTS1   LQYLQRN------------VGAQKAGGVLHLQHFPTAQLRSPHEDNSLEELTEFSK--RN  103
Zebrafish  UTS1   LQYLQR---------------SAQRAGGALHLP--------APQQDNSLEELSEFSK--RN   95
Goldfish   UTS1   LQYLQRN------------LGAQKASGVLHLPHFPAAQLRSPHEDSSLEELTEFSK--RN  103
Frog       UCN1   QREMERGDPRVQLRLSKSSLSTGHQDLVSALNVIQTAFQEGIPDESAKGFLEAFSDRLKR  116
Chicken    UCN    ---------------------GPDGPGSGVPAAEGRSEAPWPPLPAEP--------WRTRR   58
Mouse      UCN    ---------------------GVRNQGGGVRALLLLLAERFPRRAGSE-----PAGERQRR   80
Human      UCN1   ---------------------GARNQGGGARALLLLLAERFPRRAGPGRLGLGTAGERPRR   82

                       UTS1
Grass carp UTS1   DDPPISIDLTFHLLRNMIEMARNENQREQAELNRKYLDEVGK  145
Zebrafish  UTS1   DDPPISIDLTFHLLRNMIEMARIENQREQAELNRKYLDEVGK  138
Goldfish   UTS1   DDPPISIDLTFHLLRNMIEMARNENQREQAGLNRKYLDEVGK  145
Frog       UCN1   EDPPISIDLTFHILRQMIEIAKTQNQKQQAEQNRIIFDSVGK  158
Chicken    UCN    DDPPLSIDLTFHLLRHLLLLARAQSQRARADTNRRILDAVGR  100
Mouse      UCN    DDPPLSIDLTFHLLRTLLELARTQSQRERAEQNRIIFDSVGK  122
Human      UCN1   DNPSLSIDLTFHLLRTLLELARTQSQRERAEQNRIIFDSVGK  124
```

UTS1

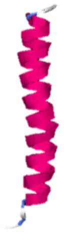

(B)

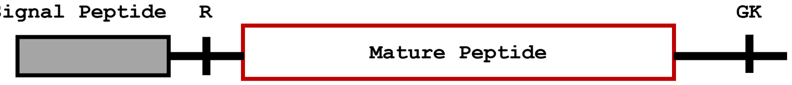


```
Grass carp UTS1   NDDPPISIDLTFHLLRNMIEMARNENQREQAELNRKYLDEV-NH2
Zebrafish  UTS1   NDDPPISIDLTFHLLRNMIEMARIENQREQAELNRKYLDEV-NH2
Goldfish   UTS1   NDDPPISIDLTFHLLRNMIEMARNENQREQAGLNRKYLDEV-NH2
Frog       UCN1   REDPPISIDLTFHILRQMIEIAKTQNQKQQAEQNRIIFDSV-NH2
Chicken    UCN    RDDPPLSIDLTFHLLRHLLLLARAQSQRARADTNRRILDAV-NH2
Mouse      UCN    RDDPPLSIDLTFHLLRTLLELARTQSQRERAEQNRIIFDSV-NH2
Human      UCN    RDNPSLSIDLTFHLLRTLLELARTQSQRERAEQNRIIFDSV-NH2
```

Supplemental Figure S3. Sequence alignment of UTS1. (A) Amino acid sequence alignment of UTS1 in different species. (B) Comparison of UTS1 mature peptide sequences in different species.

Supplemental Figure S4

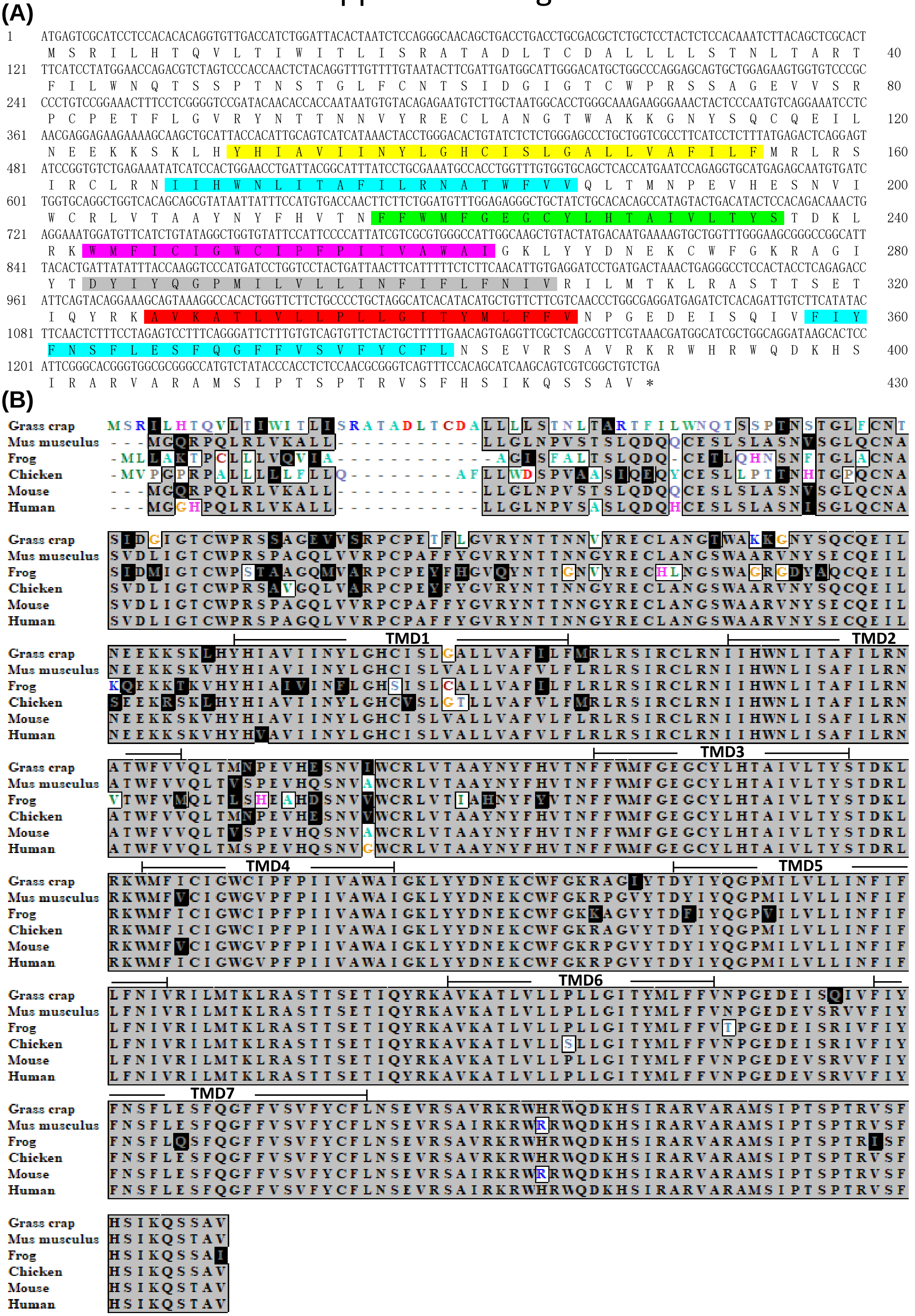

Supplemental Figure S4. Sequence alignment of CRHR1. (A) The amino acid sequence of grass carp CRHR1. The seven transmembrane regions (TMD) are represented by different color labels. (B) Sequence alignment of CRHR1 in different species

Supplemental Figure S5

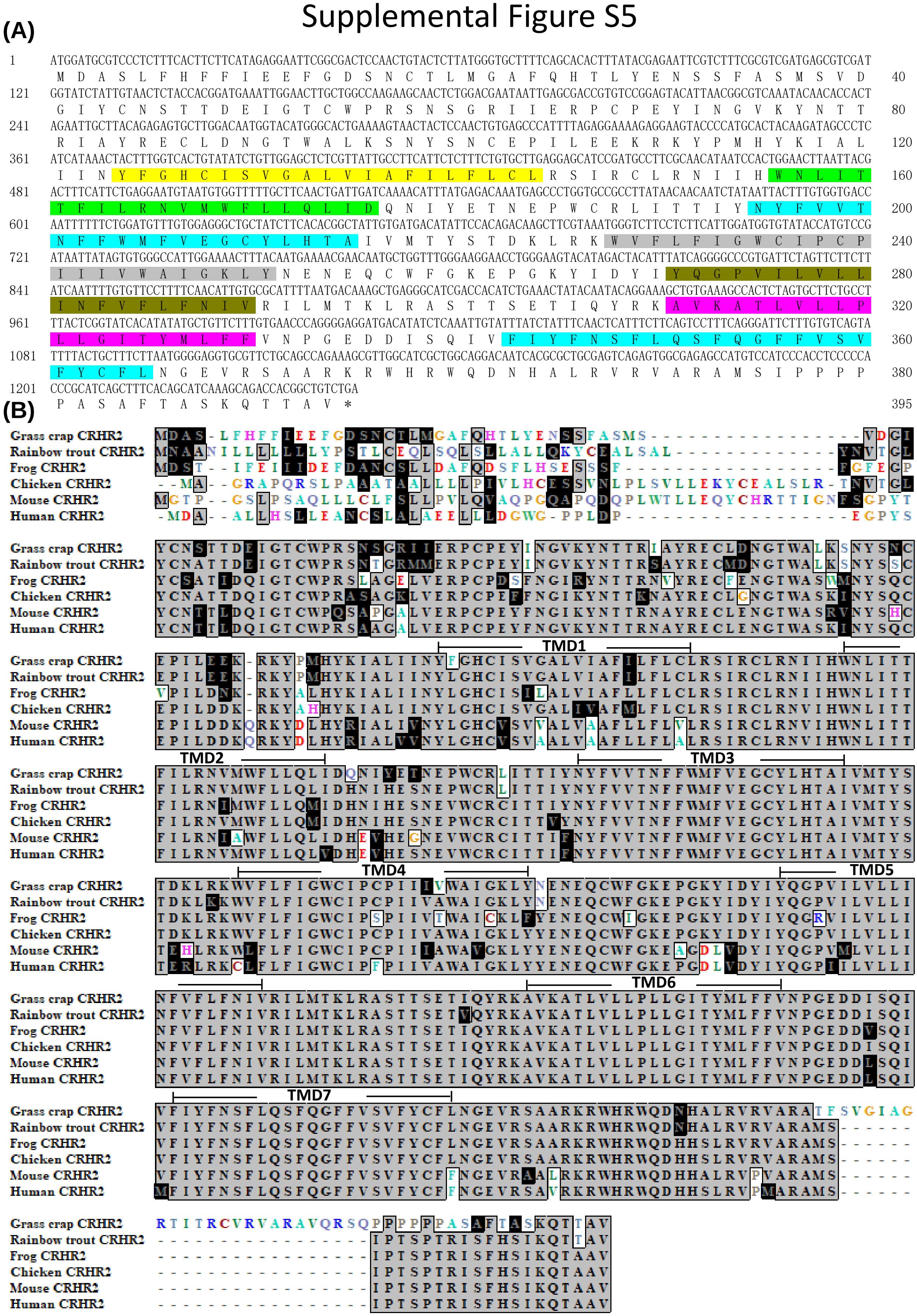

Supplemental Figure S5. Sequence alignment of CRHR1. (A) The amino acid sequence of grass carp CRHR1. The seven transmembrane regions (TMD) are represented by different color labels. (B) sequence alignment of CRHR2 in different species

Supplemental Figure S6

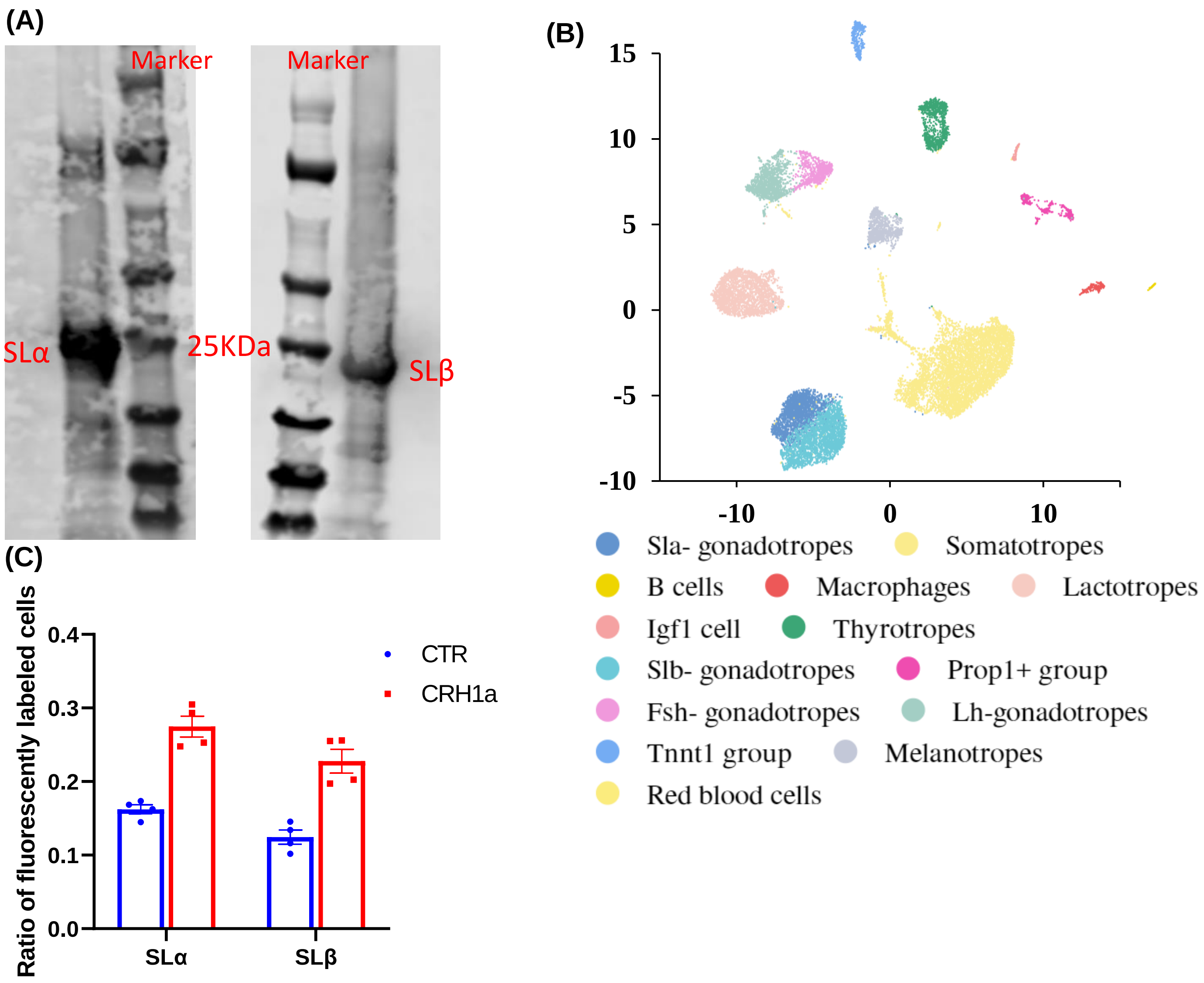

Supplemental Figure S6: Verification of the specific antibodies, immunofluorescence results and classification of distinct pituitary cell. (A) Verification of the specific antibodies (SLα and SLβ) by Western-Blot. The total protein was extracted from the pituitary tissue after fragmentation as a sample to be detected. (B) Single cell RNA sequencing (scRNA-seq) analysis of grass carp pituitary. Uniform manifold approximation and projection (UMAP) plot showing the classification of distinct cell types of grass carp pituitary. (C) At 40× magnification, ratio of cells labeled by SLα/SLβ antibody to the total number of cells in the field of view in CRH-contreated and control groups.
